# Aquila Optimization-Assisted Artificial Neural Network for Classification Problems

**DOI:** 10.3390/biomimetics11040240

**Published:** 2026-04-02

**Authors:** Gokhan Kayhan, Seyma Hasbolat Unal

**Affiliations:** Department of Computer Engineering, Ondokuz Mayis University, Kurupelit, Samsun 55139, Türkiye; seyma.hasbolat@bil.omu.edu.tr

**Keywords:** artificial neural networks, metaheuristic optimization, aquila optimizer, classification problems, machine learning

## Abstract

Artificial Neural Networks (ANNs) are models that learn patterns in input-output data. Since traditional optimization methods often get trapped in local optima when determining weight and bias values, identifying optimal parameters and enhancing network performance remain significant research areas. Heuristic algorithms are also generally used in solving optimization problems and are used to train ANNs. In the study, the parameter optimization of the ANN model was carried out using the Aquila Optimizer (AO), a recent metaheuristic algorithm, and a hybrid Aquila Optimizer optimized ANN model (AOANN) was proposed. Hybridization of algorithms contributes to the improvement of optimization performance. In this study, the proposed model was assessed on empirical datasets, including Cancer, Iris, Glass, and Wine, and its performance was compared with that of well-established ANN models. The results of the evaluation revealed that the proposed AOANN, a soft computation model, demonstrated stability in solving classification problems.

## 1. Introduction

In artificial neural networks (ANNs), a system is trained by using training data that has input values and their desired output values so that the system can learn [1]. The training consists in adjusting the connection weights so as to minimize this error of the output unit [2,3]. Different mathematical and stochastic approaches have been used to learning ANNs. It is noteworthy that the backpropagation (BP), Levenberg-Marquardt (LM) and Scaled Conjugate Gradient (SCG) are gradient based learning algorithms and most commonly used [4]. These algorithms update the connection weights by calculating and feeding back the error amount between ANN output and channeled current, from the output layer to the input layer, which aims to minimize the error based on ANN’s output [3].

The hidden layer’s activation function must be differentiable, as traditional BP algorithms involve derivative operations. These operations can be time-consuming and may get stuck in local optima in multimodal problems [5,6]. Due to these limitations, researchers have turned to metaheuristic optimization algorithms, which are more effective in global searches and stochastic approaches, to optimize ANN connection weights due to their random properties. For certain types of problems, metaheuristic algorithms have demonstrated greater effectiveness than conventional mathematical approaches in training ANNs [7].

Optimization processes aim to minimize resource usage and determine the most appropriate solution methods for reaching the final problem states [8]. Metaheuristic optimization is designed to find global solutions for problems that are difficult or impossible to solve with mathematical optimization methods, conducting fewer trials and producing results close to the exact solution within an acceptable timeframe [9,10]. These algorithms draw inspiration from the instinctive behaviors of living organisms and natural phenomena for survival [11]. They generally involve diversification (exploration) for global searches and intensification (exploitation) for local searches to find the optimum solution in the search space [12]. During the exploration phase, global results are found by scattering randomly at various points in the search space, while more precise solutions are sought in a local region during the exploitation phase. The effectiveness of the algorithms is largely influenced by how well the exploration and exploitation parameters are balanced [10]. Various hybrid methodologies have been proposed in the literature to achieve an optimal balance between these phases. For instance, the search capability of the AO has been refined by employing the GWO’s ’alpha’ position as a guiding mechanism and incorporating quasi-opposition-based learning (QOBL) throughout the process. These integrations bolster the algorithm’s stability within uncertain search landscapes, thereby facilitating high performance even in the presence of noise [13]. Alternatively, the Multi-Strategy Aquila Optimizer (MSAO) stands out in the literature as a robust approach to addressing the limitations of AO in high-dimensional spaces. By incorporating the dream-sharing strategy from the Dream Optimization Algorithm (DOA) and dynamic opposition-based learning, this method provides a reformulated equilibrium between global search and local refinement [14].

In recent years, hybrid systems combining ANNs and metaheuristic algorithms have become widespread. Literature reviews show that metaheuristic algorithms outperform traditional mathematical models in ANN weight training. The use of metaheuristic algorithms to optimize ANN parameters has accelerated over the last decade. In 2003, the Particle Swarm Optimization (PSO) was used in ANN training, achieving convergence with fewer errors and at a faster rate than the traditional BP algorithm, requiring six times fewer calculations and iterations [15]. ANN training with the Ant Colony Optimizer (ACO) for pattern classification in the medical field showed faster and lower error rates compared to traditional algorithms [16]. In 2007, the Artificial Bee Colony (ABC) algorithm optimized connection weights in signal-processing studies, outperforming PSO hybridized with an ANN and the Differential Evolution (DE) algorithm [17]. The TST metaheuristic algorithm, developed based on the Tabu Search (TS) algorithm with superior global search capability, achieved results five times faster than TS in MLP training [18]. Kattan et al. used an improved Harmony Search (HS) algorithm for MLP training, showing great success compared to traditional BP methods [19]. A neurogenetic approach combining the Genetic Algorithm (GA) and Neural Network (NN) effectively solved the Resource-Constrained Project Scheduling Problem (RCPSP), demonstrating high convergence success [20].

PSO, Imperialistic Competitive Algorithm (ICA), and traditional LM algorithms were tested in ANN training for parameter estimation affecting Stirling heat engines’ performance, with metaheuristic algorithms showing high success [21]. Aljarah et al. used the Whale Optimization Algorithm (WOA) for MLP training, finding it had a high speed of convergence and accuracy compared to six metaheuristic algorithms and BP algorithms [22]. In another study, GA was used in MLP training to determine the shortest distance in vehicle routing problems, achieving 1% convergence as the best result in the literature by testing 7 benchmark sample problems [23]. The Butterfly Optimization Algorithm (BOA) was initially utilized for training a Multi-Layer Perceptron (MLP), where it exhibited effective learning capability across various benchmark datasets(Iris, Balloon, Breast Cancer, Heart and XOR) achieving strong and consistent performance outcomes [24].

A hybrid method combining an ANN and the Cuckoo Search (CS) algorithm detected structural damages more accurately and faster than GA, PSO, and ANN algorithms alone [2]. The Jaya algorithm hybridized with an ANN correctly predicted crack lengths in engineering materials [25]. To classify medical data for disease detection, thirteen metaheuristic algorithms and gradient-based traditional LM algorithms were tested against the Equilibrium Optimization (EO) algorithm [4,11]. Hybrid metaheuristic algorithms, created by combining superior features of several metaheuristic algorithms, have become widespread for optimizing ANN weights to solve complex problems more successfully.

Tran-Ngoc et al. trained ANNs with a hybrid HGACS by combining CS and GA algorithms for damage detection in layered composite structures. This method achieved high accuracy with low computational cost due to its local search success, working parallel to the Gradient Descent (GD) method [26]. A face recognition system was developed using a Deep Neural Network (DNN) trained with a hybrid Colliding Bodies Optimization (CBO) and Multi-Verse Optimizer (MVO) algorithm, achieving an accuracy of 92.3% [27]. Alwaisi and Baykan [28] used the Shuffled Frog Leaping Algorithm (SFLA) to train ANNs for classification tasks, outperforming the conventional BP-centered algorithm in accuracy. AlRassas et al. developed the AO-ANFIS model by combining the AO algorithm and the Adaptive Neuro-Fuzzy Inference System (ANFIS) for time series forecasting in oil production, showing superior performance compared to other ANFIS hybrid methods [29]. This study used the AO metaheuristic algorithm to optimize ANN connection weights, addressing the limitations of traditional optimization methods prone to local optima. The AO metaheuristic algorithm [9] was evaluated on a set of benchmark functions and engineering problems, including 23 classical functions, 29 CEC2017 functions, 10 CEC2019 functions, and 7 engineering problems, demonstrating performance that is either superior to or comparable with other widely recognized metaheuristic methods. Additionally, empirical investigations of engineering problems showed the AO’s capability in solving real-world applications. Aarthi et al. proposed a Deep Recurrent Neural Network model supported by the Aquila Optimization Algorithm for high-accuracy, category-based classification of online shaming content [30]. Ozden and Iseri combined the COOT algorithm and ANN model, developing the COOT-ANN hybrid method with successful results [31]. Hurtado-Mora et al. proposed a hybrid neuroevolutionary framework integrating GA and NEAT to improve neural network training and mitigate the local optimum problem during the learning process, demonstrating improved classification performance [32]. Kumar et al. proposed a hybrid metaheuristic algorithm, AO-HHO, that combines the global search capability of the AO with the local exploitation of Harris Hawks Optimization(HHO) to optimize Convolutional Neural Network(CNN) hyperparameters for brain tumor classification, achieving 87.34% accuracy on a dataset of 7,023 MRI images while reducing training time to 77.85 s, outperforming conventional methods such as PSO, GA, and WOA [33]. Unlike conventional approaches that employ metaheuristic algorithms solely for weight optimization, the integration of AO into the ANN in this study constitutes an original approach by embedding the optimization mechanism directly into the learning process. The main contributions of this study are summarized as follows:

- A novel hybrid ANN model, termed AOANN, is proposed.
- The AOANN model achieves effective and robust performance across different classification datasets.
- Compared to ANN models optimized using GD based methods, AOANN reduces the likelihood of becoming trapped in local minima.
- AOANN demonstrates high efficacy in solving classification problems.
- By leveraging heuristics and stochastic processes, AOANN offers a versatile alternative to traditional gradient-based optimization methods.

## 2. Materials and Methods

This section details the fundamental working principles of traditional gradient-based learning algorithms used in the weight training of ANNs and the metaheuristic AO algorithm. It summarizes the characteristics and performance metrics of the four datasets employed in our experimental study to evaluate the AO algorithm against alternative approaches. Furthermore, the inspiration behind the proposed AOANN model, its functioning, and the optimization process of the connection parameters of ANNs are explained.

### 2.1. Datasets

In this study, the performance of the AOANN method was assessed through experiments conducted on widely used datasets, including Wine, Breast Cancer, Iris, and Glass (Table 1). These datasets represent real-world applications, although at a moderate scale. All datasets were sourced from the UCI Machine Learning Repository [34].

### 2.2. Learning Algorithms

In an ANN, many learning algorithms have working principles that are different from each other to produce the most proper output according to the input values provided to the network and optimize the connection weights to minimize error. These algorithms are used to make neural networks work in the fastest, most efficient, and most cost-effective way.

#### 2.2.1. GD Optimization Algorithm

The GD is an algorithm often used in machine-learning problems to find the local/global minimum or maximum point by starting a function’s starting point and moving gradually according to the determined step size (η). In the search for the minimum, the step direction is determined by the negative gradient of the objective function at the current position. In the GD method, the current position is updated, as in Equation (1), with each iteration by completing the derivative operation until the minimum point is reached.

(1) $$X_{n+1}=X_{n}-\eta .\frac{\partial }{\partial X_{n}}f\left(X_{n}\right)$$

When we use the GD method in an ANN, the error propagation process consists of two stages: forward calculation and backward error propagation. Forward Calculation: The forward calculation of the input vector given as an input to the system in the ANN is performed with Equations (2) and (3) ($X_{i}$ input values, $Y_{j}^{h}$ hidden layer output values, $Z_{j}$ output layer output values). The output values ($Y_{j}$) of the hidden layer neurons are represented by the forward calculation process in Equation (2) (number of hidden layers (h), number of hidden layer neurons (n), bias (b)):

(2) $$Y_{j}^{h}=f\left(\sum_{i=1}^{n}X_{i}.W_{ij}+b_{j}^{h}\right)$$

The output values ($Z_{j}$) of the output-layer neurons are represented by the forward calculation process in Equation (3) (number of output neurons (k), bias (c)):

(3) $$Z_{j}=f\left(\sum_{i=1}^{k}Y_{i}.V_{ij}+c_{j}\right)$$

The error of the network is determined as the difference between the desired output ($P_{i}$) and the current output ($Z_{i}$). The error function value ($E_{P}$) is obtained using the least-squares method from the multidimensional outputs resulting from an iteration, as Equation (4) shows.

(4) $$E_{P}=\frac{1}{2}\sum_{i=1}^{k}{\left(P_{i}-Z_{i}\right)}^{2}=\frac{1}{2}\sum_{i=1}^{k}{\left(e_{i}\right)}^{2}$$

Backward Error Propagation: The $E_{P}$ value obtained as a result of the forward calculation of the network is propagated back into the network, and the weights and bias are updated by taking the margin of error [35]. According to the GD method, weight update operations are performed as in Equations (5)–(8) (weight values ($W_{n}$), gradient of the network ($g_{n}$)):

(5) $$W_{n+1}=W_{n}+\;\;\;\Delta \;\;\;W_{n}$$

(6) $$\;\;\;\Delta \;\;\;W_{n}=-\eta .g_{n}$$

(7) $$g_{n}=\frac{\partial E_{P}}{\partial W_{n}}$$

(8) $$W_{n+1}=W_{n}-\eta .\frac{\partial }{\partial W_{n}}E_{P}\left(W\right)$$

Equation (9) shows the BP of the $E_{P}$ from the output layer to the hidden layer.

(9) $$\frac{\partial E_{P}}{\partial V_{ij}}=\frac{\partial E_{P}}{\partial Z_{j}}.\frac{\partial Z_{j}}{\partial Net_{Z_{j}}}.\frac{\partial Net_{Z_{j}}}{\partial V_{ij}}$$

Equation (10) shows the weight-update process between the hidden and the output layers.

(10) $$V_{ij(new)}=V_{ij(former)}-\eta .\frac{\partial E_{P}}{\partial V_{ij}}$$

Equation (11) shows the BP of the $E_{P}$ from the hidden layer.

(11) $$\frac{\partial E_{P}}{\partial W_{ij}}=\frac{\partial E_{P}}{\partial V_{ij}}.\left(\frac{\partial V_{ij}}{\partial Y_{j}^{h}}.\frac{\partial Y_{j}^{h}}{\partial Net_{Y_{j}}}.\frac{\partial Net_{Y_{j}}}{\partial W_{ij}}\right)$$

Equation (12) shows the weight-update process between the input and hidden layers.

(12) $${W_{ij}}_{\left(new\right)}={W_{ij}}_{\left(former\right)}-\eta .\frac{\partial E_{P}}{\partial W_{ij}}$$

The learning parameter (η) value used in the weight updates determines the optimal step length at the optimum point of finding. Choosing steps that are too small may cause the processes to be too slow, and choosing steps that are too large may cause a deviation from the result, bypassing the optimum point. The learning coefficient value is determined according to the problem type.

#### 2.2.2. LM Optimization Algorithm

The LM algorithm is an optimization method that is often used to find an objective function’s minimum point and reduces the processing load of the Newton optimization algorithm. Equation (13) shows the convergence process of Newton’s algorithm to the optimum [36].

(13) $$X_{n+1}=X_{n}-H_{n}^{-1}.\frac{\partial }{\partial X_{n}}f\left(X_{n}\right)$$

In the LM algorithm, the simplified version of the Jacobian matrix (J) (see Equation (19)) is used instead of the Hessian matrix (H), which has a smaller processing load (see Equation (14)).

(14) $$H=J^{T}*J$$

The LM algorithm uses Equations (15)–(18) to update connection weights in the training of an ANN [36] (weight values ($W_{n}$), gradient of the network ($g_{n}$), error value ($e_{n}$)):

(15) $$g_{n}=J^{T}.e_{n}$$

(16) $$\;\;\Delta \;\;\;W_{n}=-{\left(J^{T}*J\right)}^{-1}.g_{n}$$

(17) $$W_{n+1}=W_{n}+\;\;\;\Delta \;\;\;W_{n}$$

(18) $$W_{n+1}=W_{n}-{\left(J^{T}*J\right)}^{-1}.\left(J^{T}.e_{n}\right)$$

(19) $$J=\left[\begin{array}{cccc} \frac{\partial e_{11}}{\partial W_{1}} & \frac{\partial e_{11}}{\partial W_{2}} & \cdots & \frac{\partial e_{11}}{\partial W_{N}}\\ \vdots & \vdots & \ddots & \vdots \\ \frac{\partial e_{1M}}{\partial W_{1}} & \frac{\partial e_{1M}}{\partial W_{2}} & \cdots & \frac{\partial e_{1M}}{\partial W_{N}}\\ \vdots & \vdots & \ddots & \vdots \\ \frac{\partial e_{P1}}{\partial W_{1}} & \frac{\partial e_{P1}}{\partial W_{2}} & \cdots & \frac{\partial e_{P1}}{\partial W_{N}}\\ \vdots & \vdots & \ddots & \vdots \\ \frac{\partial e_{PM}}{\partial W_{1}} & \frac{\partial e_{PM}}{\partial W_{2}} & \cdots & \frac{\partial e_{PM}}{\partial W_{N}} \end{array}\right]$$

To improve the LM algorithm’s performance, Equation (20) is used instead of the Hessian matrix.

(20) $$H=(J^{T}*J)+\;\;\;\mu .I$$

I is the unit matrix, and μ is the Marquardt parameter. The μ parameter is a significant scalar value for the LM algorithm. This value takes variable values between 0 and 1, allowing LM to use the power of two algorithms. When the value is close to zero, the Newton algorithm acts as the gradient-descent algorithm when a large number is selected [37].

#### 2.2.3. SCG Optimization Algorithm

The SCG algorithm, which is created by combining the classical conjugate gradient (CG) algorithm and some features of the LM algorithm, is an optimization algorithm that performs a search according to the conjugate directions to find the optimum point of the objective function in nonlinear problem solving [38,39]. In the SCG algorithm used to optimize an ANN, iterative weighting is used for the search direction by scaling the conjugate of the direction determined in the previous step and the second-degree derivative of the error function (Hessian matrix) as the step size [40]. The search direction and step size change with each iteration and are therefore not fixed [38]. The SCG algorithm updates connection weights in the training of an ANN using Equations (21)–(31), as described in [38,41]. The following symbols are used in the equations: $W_{n}$, the weight values of the network; $\Delta W_{n}$, the weight change; $g_{n}$, the gradient of the network (direction of steepest descent); $\alpha_{n}$, the step size; $D_{n}$, the search direction (conjugate vector); $\sigma_{n}$, the weight change for the second derivative; $\lambda_{n}$, the parameter for editing Hessian uncertainty; and $E_{P}$, the error function. The weight change is calculated [42] by the scalar product of the step size and the search direction (see Equations (21) and (22)).

(21) $$W_{n+1}=W_{n}+\;\;\;\Delta \;\;\;W_{n}$$

(22) $$\;\;\Delta \;\;\;W_{n}=\alpha_{n}.D_{n}$$

The derivative of the $E_{P}$ with respect to the $W_{n}$ weights is calculated to determine $g_{n}$ (as described in Equation (23)).

(23) $$g_{n}=-\nabla E_{P}\left(W_{n}\right)=-{E\prime }_{P}\left(W_{n}\right)$$

Initially, the direction vector, $D_{0}$, is determined as the negative gradient of the $E_{P}$ (see Equation (24)). In other words, the algorithm starts searching in the steepest descent direction.

(24) $$D_{0}=-\nabla E_{P}\left(W_{0}\right)=-g_{0}$$

In the first iteration, a search is performed in the $D_{0}$ direction. The next search direction, $D_{n}$, is calculated to be the conjugate of the earlier search direction (see Equation (25)).

(25) $$D_{n}=-g_{n}+\beta_{n}*D_{n-1}$$

In the algorithm, the scaling parameters $\lambda_{n}$ and $\sigma_{n}$, which the user initially determines for scaling the Hessian matrix, are selected as in Equations (26) and (27).

(26) $$0<\sigma_{n}\leq 10^{-4}$$

(27) $$0<\lambda_{n}\leq 10^{-6}$$

The Hessian matrix approximation, $s_{n}$, is calculated as in Equation (28).

(28) $$s_{n}=\frac{{E\prime }_{P}\left(W_{n}+\sigma_{n}.D_{n}\right)-{E\prime }_{P}\left(W_{n}\right)}{\sigma_{n}}+\lambda_{n}.D_{n}$$

The step size, $\alpha_{n}$, used in the weight update, is calculated as in Equation (29).

(29) $$\alpha_{n}=\frac{{D_{n}}^{T}.g_{n}}{{D_{n}}^{T}.H.D_{n}+\lambda_{n}.{\left|\right|D_{n}\left|\right|}^{2}}$$

The $\beta_{n}$ factor used in the algorithm and the new search direction, $D_{n+1}$, are calculated as in Equations (30) and (31).

(30) $$\beta_{n}=\frac{{|g_{n+1}|}^{2}-{g_{n+1}}^{T}.g_{n}}{{g_{n}}^{T}.g_{n}}$$

(31) $$D_{n+1}=-g_{n+1}+\beta_{n}*D_{n}$$

#### 2.2.4. Aquila Optimization Algorithm

**Figure 1:**
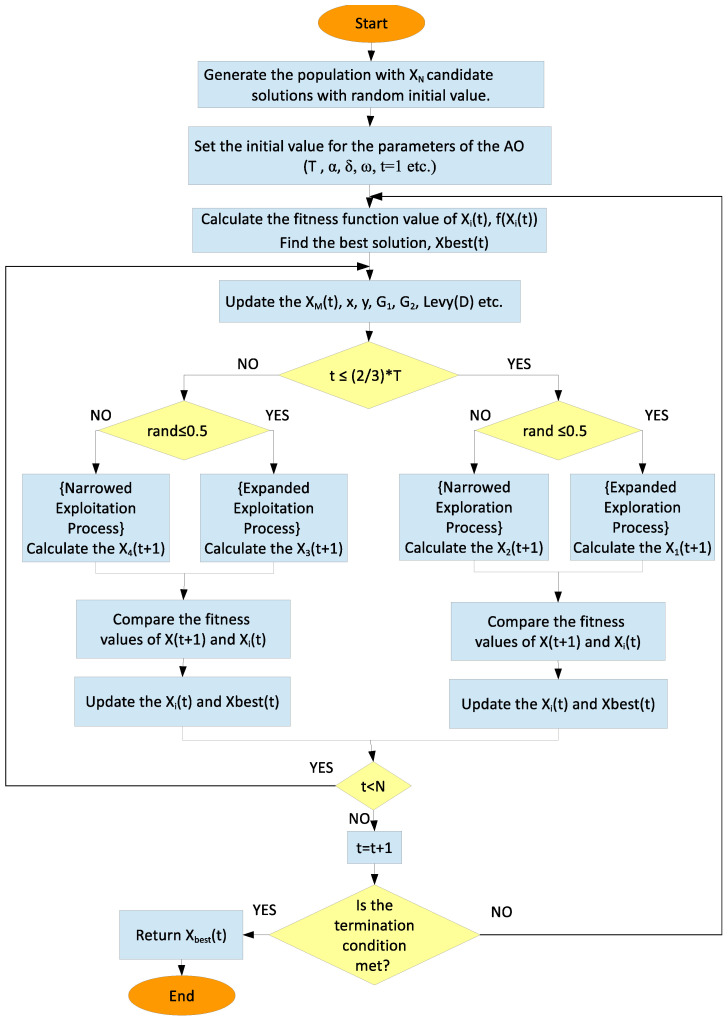
The Flowchart of the AO method.

AO is a metaheuristic optimization algorithm inspired by the Aquila’s hunting process of searching, surrounding, and attacking their prey [9]. Aquila optimization tries to find the optimum point of the objective function by following the Aquila’s four hunting steps [9,43]. Figure 1 presents the flowchart of the AO model. The population-based AO algorithm starts problem-solving with candidate solutions represented by random numbers within a specific range of limit values (UB: Upper Bound and LB: Lower Bound). The candidate solutions are initially determined using Equations (32) and (33). The candidate solutions that that make up the population with N elements are expressed as ($X_{i}$), the solution with the best fitness value that the candidate solutions have ever received are expressed as ($X_{best}$), and the population size is expressed as (D).

(32) $$X=\left[\begin{array}{cccc} X_{1,1} & X_{1,2} & \cdots & X_{1,D}\\ X_{2,1} & X_{2,2} & \cdots & X_{2,D}\\ \vdots & \vdots & \ddots & \vdots \\ X_{N,1} & X_{N,2} & \cdots & X_{N,D} \end{array}\right]$$

(33) $$X_{ij}=rand*\left(UB_{j}-LB_{j}\right)+LB_{j}$$

(34) i=1,2,…,Nj=1,2,…,D

In the AO algorithm, the positions of candidate solutions are updated at each iteration. The parameter update process consists of four consecutive phases, transitioning gradually from exploration to exploitation. Initially, the algorithm conducts a broad search to explore the solution space and then narrows the search within a focused region. In the later stages, attention shifts toward exploitation, where the search is further refined around promising areas. Let T represent the maximum number of iterations, and let t denote the current iteration index.; the exploration phase continues as long as the inequality in Equation (35) is satisfied. Once this condition no longer holds, the algorithm moves to the exploitation phase, corresponding to the local search process.

(35) $$t\leq \frac{2}{3}.T$$

#### 2.2.5. Expanded Exploration Process

The entire search region is extensively explored, and the suitability values are compared by scanning multiple regions for the optimum point. In the expanded exploration phase, the position values are updated as in Equation (36).

(36) $$X_{1}\left(t+1\right)=X_{\mathrm{best}}\left(t\right)\left(1-\frac{t}{T}\right)+\left(X_{M}\left(t\right)-X_{\mathrm{best}}\left(t\right)\right)\cdot \mathrm{rand}$$

The rand value represents the random numbers between 0–1, and the $X_{M}$ value represents the average value of the positions of all candidate solutions calculated in t iterations. It is calculated as in Equation (37).

(37) $$X_{M}\left(t\right)=\frac{1}{N}\sum_{i=1}^{N}X_{i}\left(t\right)$$

(38) ∀j=1,2,3,…,D

#### 2.2.6. Narrowed Exploration Process

Exploration continues in a region that was narrowed in the earlier method in the search space. Spiral movements are made toward the optimum point [9]. In the narrowed exploration phase, the position values are updated as in Equation (39).

(39) $$X_{2}\left(t+1\right)=X_{\mathrm{best}}\left(t\right)\cdot \mathrm{Levy}\left(D\right)+X_{R}\left(t\right)+\left(y-x\right)\cdot \mathrm{rand}$$

The x and y values representing the spiral movements and the Levy flight equation are used at this stage. $X_{R}$(t) represents a randomly selected candidate solution in the $t^{th}$ iteration. Levy flight is calculated as in Equation (40).

(40) $$Levy\left(D\right)=s*\frac{u*\sigma }{{\left|v\right|}^{\frac{1}{\beta }}}$$

The u and v values are randomly selected between 0 and 1, and the s value is set as 0.01. The σ value is calculated as in Equation (41).

(41) $$\sigma =\frac{\;\;\;\Gamma \;\;\;\left(1+\beta \right)*sin\frac{\;\;\;\pi \;\;\;\beta }{2}}{\;\;\;\Gamma \;\;\;\left(\frac{1+\beta }{2}\right)*\beta *2^{\left(\frac{\beta -1}{2}\right)}}$$

β is a fixed number with a value of 1.5. The calculation of the x and y values, which provide convergence to the optimum point by making spiral movements, is made using Equations (42)–(46).

(42) x=r∗cosθ

(43) y=r∗sinθ

(44) $$r=r_{1}+U*D_{1}$$

(45) $$\theta =-\omega *D_{1}+{\;\;\;\theta \;\;\;}_{1}$$

(46) $${\;\;\;\theta \;\;\;}_{1}=\frac{3*\pi }{2}$$

$r_{1}$ is a number from 1 to 20 that represents the number of cycles, U is a fixed number with a value of 0.00565, $D_{1}$ is an integer from 1 to problem size D, and ω is a fixed number with the value of 0.005.

#### 2.2.7. Expanded Exploitation Process

As a result of the compression of the target solution into a narrow area, a detailed local search is started in that area. In the expanded exploitation phase, the position values are updated as in Equation (47).

(47) $$X_{3}\left(t+1\right)=\left(X_{\mathrm{best}}\left(t\right)-X_{M}\left(t\right)\right)\cdot \alpha -\mathrm{rand}+\left((UB-LB)\cdot \mathrm{rand}+LB\right)\cdot \delta$$

The parameters α and δ have a fixed value as small as 0.1.

#### 2.2.8. Narrowed Exploitation Process

Local search operations are performed for the possible optimum solution, which is compressed into an exceedingly small region in the search space. In the narrowed exploitation phase, the position values are updated as in Equation (48).

(48) $$X_{4}\left(t+1\right)=QF\cdot X_{\mathrm{best}}\left(t\right)-G_{1}\cdot X\left(t\right)\cdot \mathrm{rand}-G_{2}\cdot \mathrm{Levy}\left(D\right)+\mathrm{rand}\cdot G_{1}$$

At this stage, the QF value, which is the quality function used to ensure balance in the exploration and exploitation processes; the $G_{1}$ value, which is used to determine random exploitation movements in the candidate solution’s immediate vicinity; and the $G_{2}$ value, which decreases from 2 to 0, indicating the flight slope, are used in the method to track the best solution from the first position to the last position. QF, $G_{1}$, and $G_{2}$ values are calculated as in Equations (49)–(51).

(49) $$QF\left(t\right)=t^{\frac{2*rand-1}{{\left(1-T\right)}^{2}}}$$

(50) $$G_{1}=2*rand-1$$

(51) $$G_{2}=2*\left(1-\frac{t}{T}\right)$$

In the AO algorithm, the best solution value ($X_{best}$), as a result of all iterations, is the solution to the problem. The Aquila optimization algorithm’s processing steps [29,43] are given in Algorithm 1.
**Algorithm 1** AO Algorithm  1:Set the parameters (T, α,δ,ω, etc.) and generate random population, $X_{N}$  2:**while** (termination condition not met) **do**  3:    Calculate the fitness function value of $X_{i}$(t) and find the $X_{best}$(t)  4:    **for** (i=1,2, …, N) **do**  5:        Calculate the $X_{M}$(t) and update parameters (x,y,G1,Levy(D) etc.)  6:        **if** $\left(t\leq \frac{2}{3}*T\right)$ **then**  7:           **if** rand≤0.5 **then**  8:               Expanded Exploration: $X_{i}\left(t\right)\overset{\mathrm{Equation}\;(36)}{\to }X_{1}\left(t+1\right)$  9:               **if** f($X_{1}$(t+1)) < f($X_{i}$(t)) **then**10:                   $X_{i}$(t) ← $X_{1}$(t+1)11:                   **if** f($X_{1}$(t+1)) < f($X_{best}$) **then** $X_{best}$ ← $X_{1}$(t+1)12:                   **end if**13:               **end if**14:           **else**15:               Narrowed Exploration: $X_{i}\left(t\right)\overset{\mathrm{Equation}\;(39)}{\to }X_{2}\left(t+1\right)$16:               **if** f($X_{2}$(t+1)) < f($X_{i}$(t)) **then**17:                   $X_{i}$(t) ← $X_{2}$(t+1)18:                   **if** f($X_{2}$(t+1)) < f($X_{best}$) **then** $X_{best}$ ← $X_{2}$(t+1)19:                   **end if**20:               **end if**21:           **end if**22:        **else**23:           **if** rand≤0.5 **then**24:               Expanded Exploitation: $X_{i}\left(t\right)\overset{\mathrm{Equation}\;(47)}{\to }X_{3}\left(t+1\right)$25:               **if** f($X_{3}$(t+1)) < f($X_{i}$(t)) **then**26:                   $X_{i}$(t) ← $X_{3}$(t+1)27:                   **if** f($X_{3}$(t+1)) < f($X_{best}$) **then** $X_{best}$ ← $X_{3}$(t+1)28:                   **end if**29:               **end if**30:           **else**31:               Narrowed Exploitation: $X_{i}\left(t\right)\overset{\mathrm{Equation}\;(48)}{\to }X_{4}\left(t+1\right)$32:               **if** f($X_{4}$(t+1)) < f($X_{i}$(t)) **then**33:                   $X_{i}$(t) ← $X_{4}$(t+1)34:                   **if** f($X_{4}$(t+1)) < f($X_{best}$) **then** $X_{best}$ ← $X_{4}$(t+1)35:                   **end if**36:               **end if**37:           **end if**38:        **end if**39:    **end for**40:**end while**41:Return $X_{best}$

#### 2.2.9. Proposed AOANN Model

**Figure 2:**
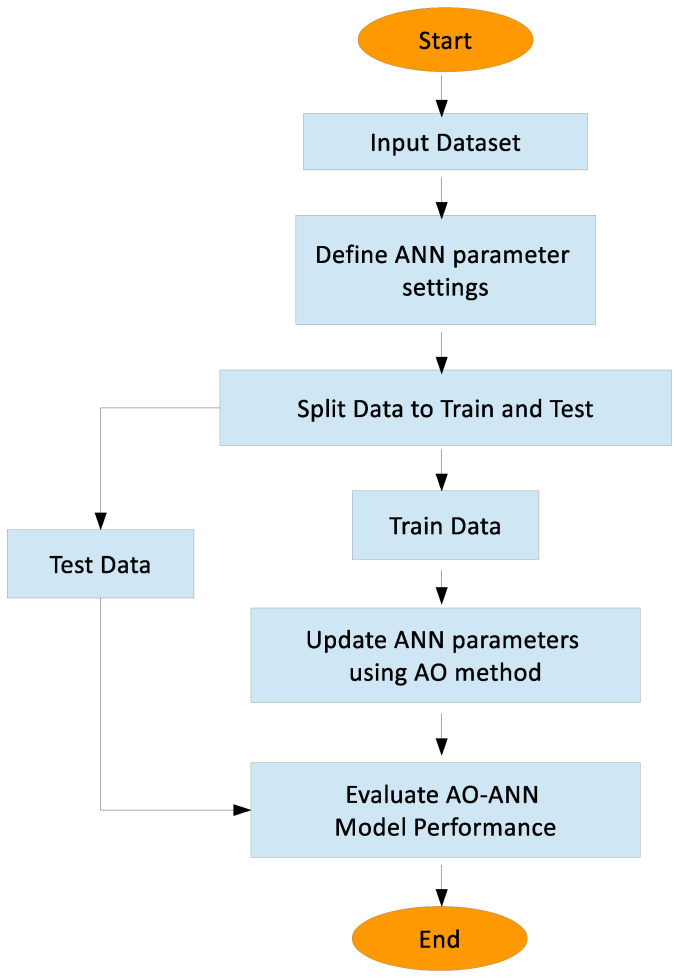
The Flowchart of the Proposed AOANN Model.

Gradient-based optimization algorithms are computationally efficient but are prone to getting stuck in local minima. On the other hand, metaheuristic algorithms often have higher computational complexity, but they have the advantage of avoiding local minima by searching a wider solution space. Gradient-based algorithms optimize model parameters by iteratively updating them according to the gradient of the loss function with respect to those parameters. However, when the loss function has multiple local minima, these algorithms can get stuck in a suboptimal solution. Metaheuristic algorithms, on the other hand, employ a more diverse search strategy, often guided by heuristics, to find the global optimum solution. They do not rely solely on the gradient of the loss function, and therefore can explore regions of the solution space that gradient-based algorithms may miss. The AO, a powerful metaheuristic approach, has been employed to optimize the parameters of ANNs, leveraging the exploration and exploitation capabilities inherent in metaheuristic algorithms. The developed AOANN hybrid model ensures that the ANN used in problem solving approaches the solution in the fastest way possible with fewer errors and produces accurate and consistent results. Figure 2 shows the flowchart of the AOANN model. Algorithm 2 presents the processing steps of the developed AOANN.
**Algorithm 2** Proposed AOANN Algorithm  1:Inputs: Dataset, Number of candidate solutions (N) and max. iteration(T)  2:Build the ANN network and define ANN parameter settings  3:Generate a random population of N candidate solutions ($X_{ND}$) (D is dimension size of the solutions)  4:Set the parameters of the AO  5:Split Data to Train and Test  6:t ← 1  7:**while** t≤T **do**  8:    Determine the fitness function value of each $X_{i}$(t)  9:    Find the best solution, $X_{best}$ (t)10:    Update ANN parameters using AO method11:    t ← t+112:**end while**13:Return $X_{best}$14:Evaluate AOANN Model Performance

#### 2.2.10. Complexity Analysis of the AOANN Algorithm

This section provides a detailed complexity analysis of the AO algorithm utilized for parameter optimization in ANNs. Comprehending this complexity is pivotal for accurately assessing the computational requirements and performance efficacy of the optimization process.

**Initialization Phase:** During the initialization phase, the following operations are performed:

- Data and Parameter Initialization: The process of initially setting up the dataset and the ANN parameters typically exhibits a complexity of O(B); this implies that the computational cost of the operation is proportional to the network’s construction complexity, denoted as *B*.
- Random Population Generation: The process of creating a random initial population consisting of *N* possible solutions (candidate solutions), each with dimension *D* has a complexity of O(N×D).
- AO Parameters and Data Splitting: The processes of determining the fixed parameters of the AO algorithm and splitting the dataset into training and testing groups generally possess a constant time complexity of O(1).

The total complexity for the initialization phase is:

O(B)+O(N×D)+O(M)

where *M* denotes the size of the dataset.

**Main Optimization Loop:** In the main optimization loop, the following operations are carried out:

- Fitness Function Evaluation: Calculating the fitness value of every candidate solution in the population has a complexity of O(N×D).
- Best Solution Determination: Identifying the solution with the highest performance among all candidate solutions is typically executed with a complexity of O(N).
- Updating ANN Parameters Using AO: Renewing the ANN parameters using the AO algorithm incurs a complexity cost of O(N×D) for each iteration.
- Incrementing Iteration Counter: The operation of advancing the iteration counter by one unit has a complexity of O(1), as it is a constant-time operation.

The total complexity for the main optimization loop across *T* iterations is:

$$T\times \left(O(N\times D)+O(N)+O(N\times D)\right)=O\left(T\times N\times D\right)$$

where *T* denotes the maximum number of iterations.

**Final Steps:** The final steps include:

- Returning the Best Solution: This operation has a complexity of O(1).
- Evaluating Model Performance: The performance evaluation of the AOANN model (ANN optimized by the AO) has a complexity of O(E), where (E) is specific to the evaluation method and test conditions.

**Overall Complexity Analysis:** Combining all phases, the overall time complexity is:

O(B)+O(N×D)+O(M)+O(T×N×D)+O(E)

where

- O(B): Complexity of setting up the ANN;
- O(N×D): Complexity of generating the initial population and fitness evaluations;
- O(M): Complexity of data splitting;
- O(T×N×D): Complexity of the main optimization loop;
- O(E): Complexity of performance evaluation.

For large values of *T*, *N*, and *D*, the term O(T×N×D) dominates the overall complexity. This indicates that the AO algorithm significantly impacts the complexity and is the primary factor in the computational requirements of the optimization process.

The overall complexity grows proportionally with the iteration count, the number of candidate solutions, and the solution dimension. The AO algorithm, being the most complex part of the process, is the key determinant of the optimization complexity, overshadowing other stages.

#### 2.2.11. Performance Metrics

In classification problems, the confusion matrix (Table 2) is often used in testing the system with the connection weights obtained from the training. Multiple evaluation metrics can be calculated with this matrix, which shows the possible situations of the actual value and the estimated value in a dataset classified as positive and negative.

True Positive (TP) refers to instances correctly identified as positive, while True Negative (TN) represents cases accurately recognized as negative. In contrast, a False Positive (FP) arises when a negative sample is mistakenly classified as positive, whereas a False Negative (FN) occurs when a positive sample is incorrectly classified as negative [44]. Metric of Accuracy (ACC) is calculated [45,46,47] as the ratio of correctly predicted values to the number of all predictions (see Equation (52)).

(52) $$\mathrm{ACC}=\frac{\mathrm{TP}+\mathrm{TN}}{\mathrm{TP}+\mathrm{FP}+\mathrm{TN}+\mathrm{FN}}$$

The F1-score is a performance evaluation metric obtained by computing the harmonic mean of the Recall and Precision values derived from the confusion matrix. It ranges from 0 to 1, where values approaching 1 signify a higher likelihood of model success [45,46,48]. The calculation of the F1-score is expressed in Equation (53) [47].

(53) $$F1\text{-}\mathrm{score}=2\cdot \frac{\mathrm{Recall}\cdot \mathrm{Precision}}{\mathrm{Recall}+\mathrm{Precision}}$$

Recall indicates how much of the data determined as positive is predicted as such [45,46]. It is calculated [47] as in Equation (54).

(54) $$\mathrm{Recall}=\frac{\mathrm{TP}}{\mathrm{TP}+\mathrm{FN}}$$

Precision indicates how often the positively predicted values are actually positive [45,46]. It refers to the quality of the classification process [44]. It is calculated [47] as in Equation (55).

(55) $$\mathrm{Precision}=\frac{\mathrm{TP}}{\mathrm{TP}+\mathrm{FP}}$$

The CE is an evaluation method that measures the difference between probability distributions belonging to different sets of events and allows for the measurement of model performance by being preferred as an error function in classification problems [49]. In an ANN with K classes and N data, the CE value between the actual output (T) and the predicted output (Y) is calculated [50] as in Equation (56).

(56) $$\mathrm{CE}=-\sum_{n=1}^{N}\sum_{i=1}^{K}T_{ni}\cdot \log\left(Y_{ni}\right)$$

Receiver Operating Characteristic (ROC) represents the curves drawn between the points (0, 0) and (1, 1) as a result of showing the FP-Rate (see Equation (57)) and TP-Rate (see Equation (58)) on the x- and y-axis, respectively. It is often preferred in machine learning (ML) to evaluate models’ performance. The fact that the TP rate is high and the FP rate is low indicates that the models’ performance is high [51].

(57) $$\text{FP-Rate}\;\left(\mathrm{FPR}\right)=\frac{\mathrm{FP}}{N}=\frac{\mathrm{FP}}{\mathrm{TN}+\mathrm{FP}}$$

(58) $$\text{TP-Rate}\;\left(\mathrm{TPR}\right)=\frac{\mathrm{TP}}{P}=\frac{\mathrm{TP}}{\mathrm{TP}+\mathrm{FN}}$$

The Receiver Operating Characteristic Area Under the Curve (AUC) is used to evaluate the performance of classifier models and takes values between 0 and 1. A value close to 1 indicates that the model’s predictions are highly accurate, a value of 0.5 indicates that the predictions are randomly distributed, and values close to 0 indicate that the predictions are made in the wrong direction [51].

Cohen’s kappa (CK) is an evaluation method that shows the harmony between the actual output and the predicted output. Kappa takes values between −1 and +1. The fact that the kappa value is close to +1 in the model indicates that the randomness is low and that the harmony and success are high [52]. CK score is calculated as in Equations (59)–(63).

(59) $$\mathrm{CK}=\frac{P_{O}-P_{E}}{1-P_{E}}$$

(60) $$P_{O}=\frac{\mathrm{TP}+\mathrm{TN}}{\left(\mathrm{TP}+\mathrm{FN}+\mathrm{FP}+\mathrm{TN}\right)}$$

(61) $$d=\frac{\left(\mathrm{TN}\mathrm{FP}).(\mathrm{TN}+\mathrm{FN}\right)}{{\left(\mathrm{TP}+\mathrm{FN}+\mathrm{FP}+\mathrm{TN}\right)}^{2}}$$

(62) $$y=\frac{\left(\mathrm{FN}+\mathrm{TP}).(\mathrm{FP}+\mathrm{TP}\right)}{{\left(\mathrm{TP}+\mathrm{FN}+\mathrm{FP}+\mathrm{TN}\right)}^{2}}$$

(63) $$P_{E}=d+y$$

## 3. Experimental Results

In this paper, a new hybrid ANN model with the AO was proposed and evaluated over classification datasets. The effectiveness of the proposed AOANN algorithm was tested by comparing it to the Levenberg-Marquardt optimized ANN (LMANN), Scaled Conjugate Gradient optimized ANN (SCGANN), and Gradient Descent optimized ANN (GDANN) algorithms. Experiments were performed using an Intel i5 processor using a 3.2 GHz clock and 8 GB RAM, while the models were implemented using MATLAB 2021 software. The model had a single hidden layer, and the neuron number at this layer was selected based on a trial-and-error methodology. Hence, 10 neurons were found as enough for the Cancer, Glass, and Iris datasets in the models; for the Wine dataset, three neurons were enough. During the training of the AOANN model, 100,000 iterations, LB = −5, UB = 5, and a population of 20 were fixed as parameter values. These are parameter values used at the initialization of the SCGANN, LMANN, and GDANN models and running them for 1000 epochs. All these parameters were determined by trial and error. Metaheuristics are iterative search processes conducted on data towards the minimization of any objective function. Each iteration might be defined as a step in the search and hence involves a movement from one point to another; thus, it is different from epoch by the step carried out. The performance of the novel model was evaluated using four datasets: Wine, Cancer, Iris, and Glass. The rigorous evaluation included the K-Fold validation scheme with 5 folds throughout the entire training and subsequent testing procedure. The performances of the algorithms used for neural network training were measured by two primary metrics, ACC and Cross-Entropy (CE). Additionally, the models’ test-phase performances were compared based on several complementary measures of evaluation, including the CK, F1-score, Receiver Operating Characteristic–Area Under the Curve (ROC-AUC), and Confusion Matrix. For each model, the average performance on its corresponding dataset was computed by taking the arithmetic mean of the CE and ACC values obtained across all folds. The aggregated results of these evaluations are shown in Table 3.

The aim of the model training was CE, as generally it is preferred as a standard objective function in classification problems. By minimizing the CE, one gets better classification; hence, the lower the value, the better the performance, and a value of zero corresponds to no error. ACC is a metric that expresses the ratio of correctly predicted samples with respect to the total number of instances in the dataset, including true positives and true negatives. Its value varies between 0 and 1, where values closer to 1 correspond to superior classification performances.

As seen from Table 3, the AO method performed better compared to the other methods evaluated in this study. More specifically, the AOANN model performed with accuracy rates of 98.32% on the Wine dataset, 95.42% on the Cancer dataset, and 94.41% on the Glass dataset. On the Iris dataset, the highest test accuracy of 95.33% was achieved by both the AOANN and SCGANN models. The minimum CE values obtained during the training and test phases verify that the classification was done in a consistent and realistic manner. This observation can be seen more clearly in Table 4, which presents the results of a representative sample fold. In addition to the ACC and CE metrics, the F1-score further justifies the proposed method, since the F1-score provides a more appropriate evaluation of the classification performance, especially in datasets with an imbalanced class distribution. Table 4 depicts the performance measures obtained on a randomly selected fold during the training phase, and thus allows for a comparative evaluation of the test data based on fold-wise ACC, F1-score, CK, and CE metrics. An examination of the F1-score and CK metrics along with ACC and CE results provides a better understanding of the performance of the proposed method, since these metrics are more informative, especially in multi-class and imbalanced datasets. Furthermore, the AUC, which refers to the area under the ROC curve, is widely recognized as a basic indicator of the effectiveness of any classifier. An AUC value of 1.0 corresponds to the best possible classification. Based on the test data given in Table 4, it can be noted that

- For the Wine dataset, the AOANN model demonstrated optimal performance, achieving the highest metrics across test ACC, CE, F1-score, and CK.
- On the Cancer dataset, while AOANN and GDANN exhibited identical test ACC values, the AOANN model yielded superior results for both the F1-score and CK metrics.
- The analysis of the Iris dataset indicated that both the AOANN and GDANN models achieved the maximum recorded values for test ACC, F1-score and CK.
- Furthermore, for the Glass dataset, the AOANN model once again produced the most favorable performance across all evaluated metrics: test ACC, CE, F1-score and CK.

**Figure 3:**
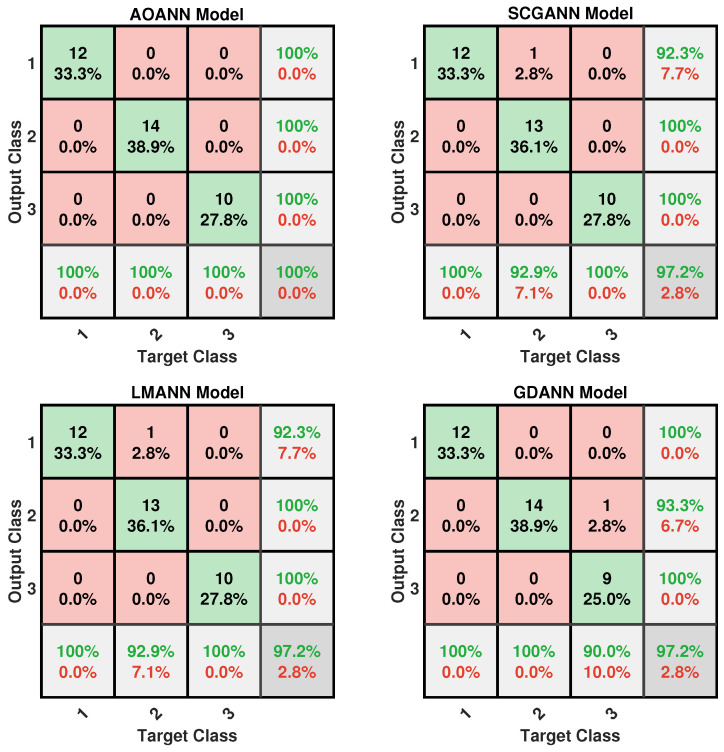
Confusion matrix for the Wine test dataset across the evaluated models.

**Figure 4:**
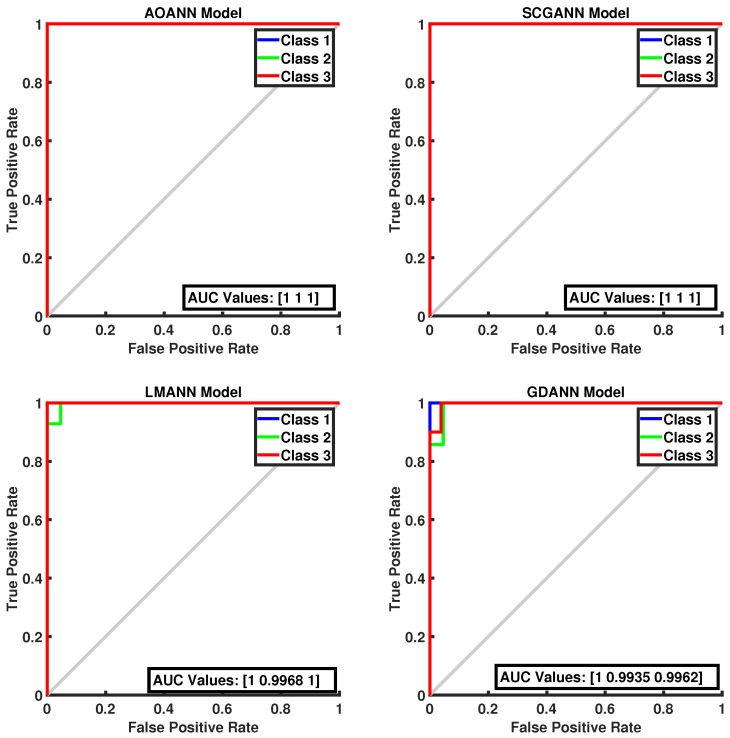
ROC Curve for the Wine test dataset across the evaluated models.

**Figure 5:**
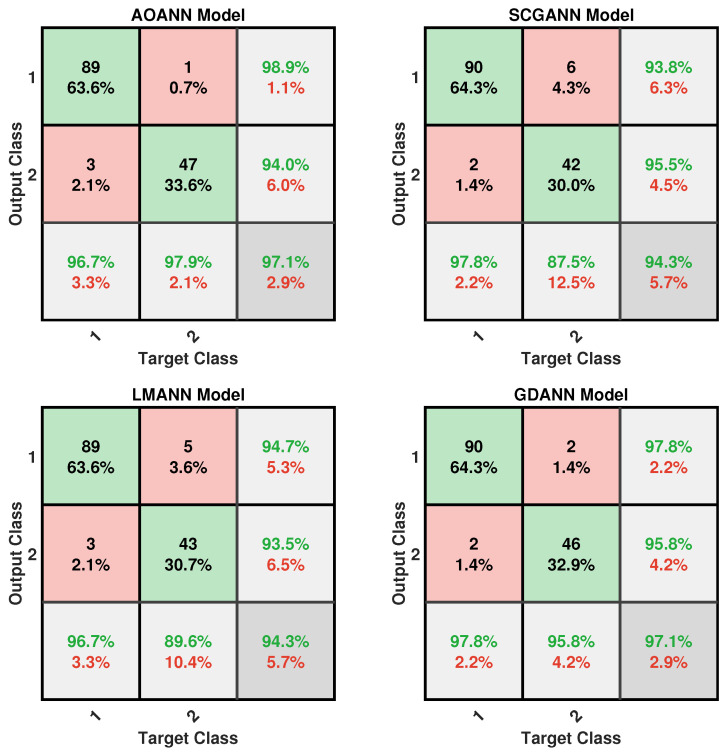
Confusion matrix for the Breast Cancer test dataset across the evaluated models.

**Figure 6:**
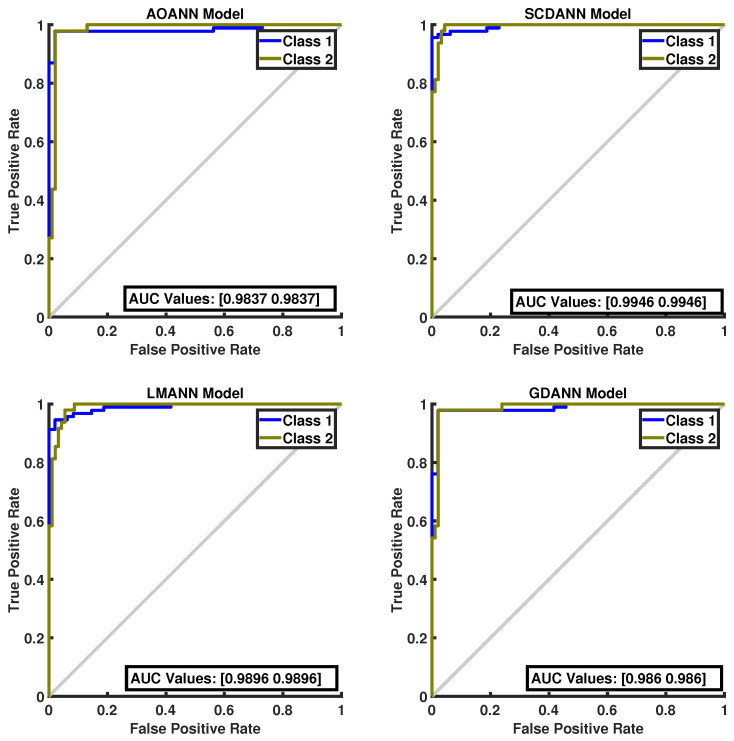
ROC Curve for the Breast Cancer test dataset across the evaluated models.

**Figure 7:**
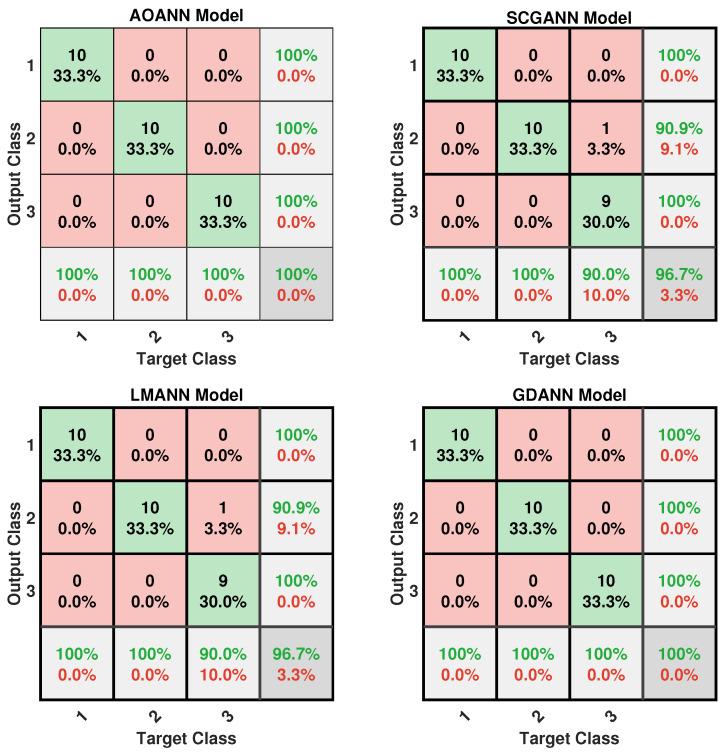
Confusion matrix for the Iris test dataset across the evaluated models.

The classification performance of the proposed AOANN model on the Wine dataset, which contains three classes, is confirmed through the confusion matrix presented in Figure 3. The matrices also highlight the classification inaccuracies of the alternative models. Specifically, the SCGANN and LMANN networks incorrectly labeled some class 2 instances as class 1, whereas the GDANN model confused certain class 3 samples with class 2. As illustrated in Figure 4, which shows the ROC curves and AUC values for all methods, the AOANN and SCGANN models demonstrate superior performance, while GDANN performs the weakest. Because the Wine dataset is balanced, the F1-score metric effectively reflects classification success. Likewise, the AOANN model’s classification accuracy on the Cancer dataset (a binary classification task) is validated by the confusion matrix presented in Figure 5. This matrix also discloses the error distribution among the other models: AOANN and GDANN each produced 4 misclassifications, whereas SCGANN and LMANN misclassified 8 samples. According to the ROC curves and AUC values displayed in Figure 6, AOANN shows a slightly lower AUC than the other methods. Nevertheless, due to the class imbalance in this dataset, the F1-score provides a more meaningful indicator of model performance, as the ROC curve represents an average across all thresholds. Finally, for the Iris dataset, another three-class dataset, the AOANN model’s effectiveness is validated by the confusion matrix in Figure 7. The same figure also reveals that SCGANN and LMANN misclassified several class 3 instances as class 2, indicating a consistent pattern in their classification tendencies.

**Figure 8:**
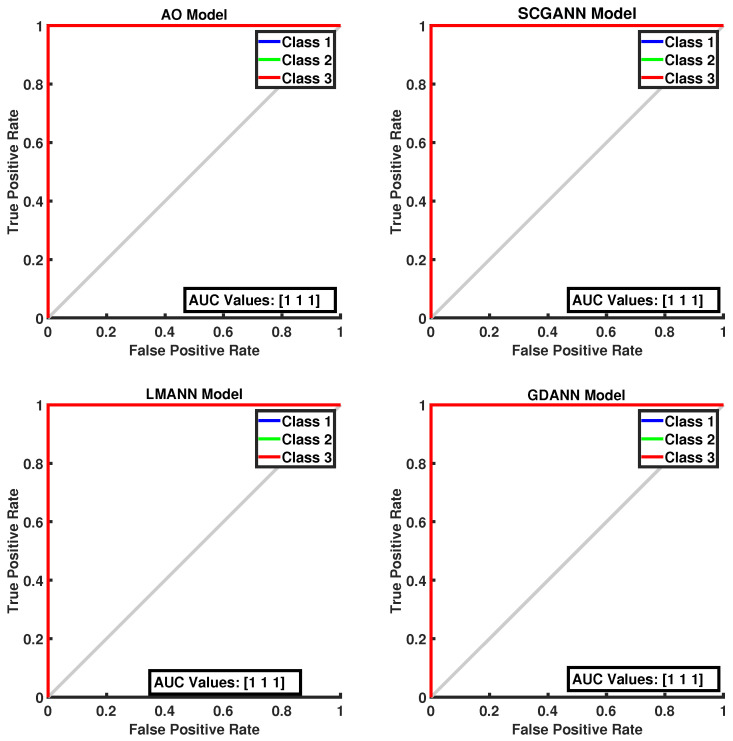
ROC Curve for the Iris test dataset across the evaluated models.

**Figure 9:**
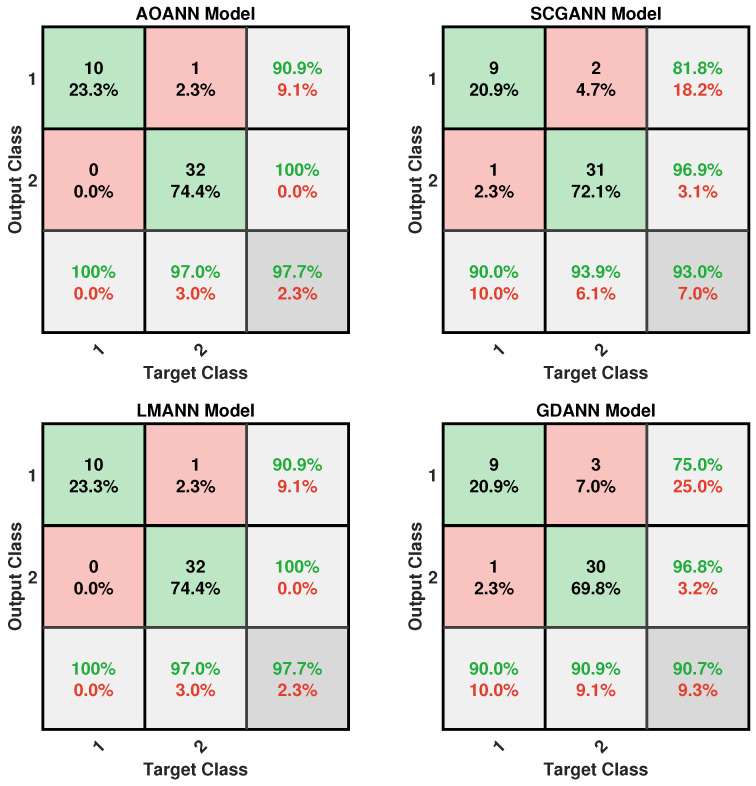
Confusion matrix for the Glass test dataset across the evaluated models.

**Figure 10:**
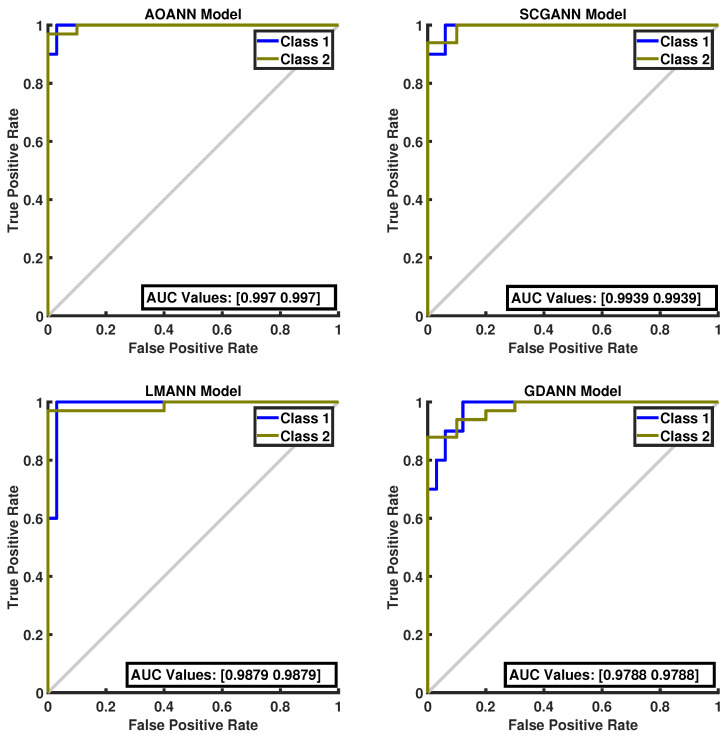
ROC Curve for the Glass test dataset across the evaluated models.

Figure 8 presents the ROC curves and corresponding AUC values for all methods evaluated on the Iris dataset. As illustrated in the figure, the proposed AOANN model exhibits classification performance comparable to that of the SCGANN, GDANN, and LMANN models. Figure 9 shows the confusion matrices of the AOANN model and the remaining models evaluated on the Glass dataset. This matrix confirms the accuracy achieved by the AOANN model on the Glass dataset, which represents a binary classification task. Moreover, it reveals the distribution of misclassifications among the competing models. Specifically, the AOANN and LMANN models each produced one incorrect classification, while SCGANN and GDANN resulted in three and four errors, respectively. Figure 10 presents the ROC curves and corresponding AUC values for all methods applied to the Glass dataset. The results clearly show that the proposed AOANN model attains the best performance, while the GDANN model exhibits the weakest classification ability.

The proposed AOANN model consistently achieves superior accuracy and lower error rates across all benchmarks. Although it requires more computational effort than gradient-based baselines, this is a deliberate trade-off justified by its ability to avoid local minima and establish stable classification boundaries. As summarized in Table 5, the synergy between the AO and ANN architecture ensures a robust training process, prioritizing solution quality over execution speed.

## 4. Conclusions and Discussion

While gradient-based algorithms are computationally efficient, metaheuristic algorithms possess the advantage of escaping local minima, making them particularly suitable for optimization problems where gradient-based methods may fail. Therefore, the trade-off between computational complexity and the ability to avoid local minima must be carefully considered when selecting an appropriate optimization algorithm for a given problem. In this study, an enhanced hybrid ANN model, referred to as AOANN, was introduced to effectively handle both binary and multi-class classification tasks. The model integrates the recently developed metaheuristic optimization algorithm, AO, for optimizing ANN parameters. The effectiveness of the proposed AOANN model was assessed using four benchmark datasets (Wine, Iris, Breast Cancer and Glass) obtained from the UCI repository and compared with well-known models such as LMANN, SCGANN, and GDANN. Experimental results revealed that AOANN achieved perfect ACC (100%) on the Iris and Wine test datasets, while attaining 97.67% and 97.14% accuracy on the Glass and Breast Cancer test datasets, respectively. Moreover, the AOANN model achieved notably high F1-score and CK values across all test datasets compared to other methods. Specifically, the F1-score and CK values were both 1.0 for the Iris and Wine datasets. For the Glass dataset, these values were 0.9695 and 0.9370, respectively, while for the Breast Cancer dataset they were 0.9687 and 0.9372. These results are consistent with the theoretical expectation that metaheuristic-based optimization improves generalization performance by enabling a better balance between exploration and exploitation, thereby leading to more robust and accurate classification outcomes. Overall, the findings demonstrate that the AOANN model outperforms other algorithms across all evaluated datasets.

In future work, the proposed model will be further developed to address regression problems and to optimize hyperparameters in deep learning architectures. Furthermore, a more comprehensive parameter sensitivity analysis could yield deeper insights into the model’s behavior. Therefore, we intend to incorporate such an investigation in future work to more thoroughly examine how different parameters influence the performance of the AOANN model. Additionally, future studies could evaluate the AOANN model on higher-dimensional datasets to further explore its scalability and performance on large-scale non-linear problems. Finally, a memetic approach integrating the AO for global exploration with gradient-based methods for local fine-tuning will be explored to further enhance the training efficiency of neural architectures.

## Figures and Tables

**Table 1:** Specification of the datasets used for classification.

DataSet Name	Samples	Features	Classes
Wine	178	13	3
Breast Cancer	699	9	2
Iris	150	4	3
Glass	214	9	2

**Table 2:** Confusion Matrix.

	Predicted Positive	Predicted Negative
Actually Positive	True Positive (TP)	False Negative (FN)
Actually Negative	False Positive (FP)	True Negative (TN)

**Table 3:** Average Evaluation Results Across Datasets (5-Fold).

		Train Data		Test Data	
Dataset	Method	ACC	CE	ACC	CE
Wine	AO	1.0000	0.0071	0.9832	0.0307
	SCG	1.0000	0.0000	0.9662	0.0429
	LM	1.0000	0.0000	0.9775	0.0118
	GD	0.9298	0.1277	0.8871	0.1426
Cancer	AO	0.9971	0.0070	0.9542	0.1415
	SCG	1.0000	0.0000	0.9370	0.3981
	LM	0.9993	0.0007	0.9384	0.0579
	GD	0.9503	0.0714	0.9428	0.0776
Iris	AO	1.0000	0.0000	0.9533	0.4577
	SCG	1.0000	0.0000	0.9533	0.2718
	LM	1.0000	0.0000	0.9333	0.0368
	GD	0.9217	0.1371	0.9333	0.1408
Glass	AO	0.9953	0.0109	0.9441	0.1179
	SCG	1.0000	0.0000	0.9349	0.3413
	LM	1.0000	0.0000	0.9394	0.0570
	GD	0.9066	0.1221	0.9158	0.1231

**Table 4:** Sample Fold Evaluation Results for the Datasets.

		Train Data		Test Data			
Dataset	Method	ACC	CE	ACC	CE	F1-Score	CK
Cancer	AO	0.9893	0.0181	0.9714	0.0919	0.9687	0.9372
	SCG	1.0000	0.0000	0.9429	0.2905	0.9363	0.8706
	LM	0.9982	0.0018	0.9429	0.0518	0.9362	0.8719
	GD	0.9481	0.0667	0.9714	0.0576	0.9680	0.9366
Wine	AO	1.0000	0.0087	1.0000	0.0147	1.0000	1.0000
	SCG	1.0000	0.0000	0.9722	0.0547	0.9753	0.9590
	LM	1.0000	0.0000	0.9722	0.0218	0.9753	0.9590
	GD	0.9366	0.1508	0.9722	0.1476	0.9721	0.9577
Iris	AO	1.0000	0.0000	1.0000	0.0000	1.0000	1.0000
	SCG	1.0000	0.0000	0.9667	0.0134	0.9681	0.9500
	LM	1.0000	0.0000	0.9667	0.0220	0.9681	0.9500
	GD	0.9500	0.1337	1.0000	0.1375	1.0000	1.0000
Glass	AO	0.9883	0.0168	0.9767	0.0494	0.9695	0.9370
	SCG	1.0000	0.0000	0.9302	0.2035	0.9063	0.8111
	LM	1.0000	0.0000	0.9767	0.0235	0.9695	0.9370
	GD	0.9123	0.0955	0.9070	0.0919	0.8812	0.7564

**Table 5:** Summary of Performance Metrics and Evolutionary Advantages of AOANN.

Metric	AOANN Advantage	Academic Significance
ACC	Highest classification rates	Superior discriminative capacity
CE	Lowest variance values	Model stability
AUC/ROC	Values nearest to 1.0	Maximum class separation power
F1-Score	Balanced precision and recall	Reliability in sensitive diagnostics

## Data Availability

Publicly available datasets were analyzed in this study. This data can be found here: UCI Machine Learning Repository [https://archive.ics.uci.edu/ml/index.php]; accessed on 10 March 2026.

## Abbreviations

The following abbreviations are used in this manuscript:

AO	Aquila Optimizer
ANN	Artificial Neural Network
AOANN	Aquila Optimizer optimized ANN
SCGANN	Scaled Conjugate Gradient optimized ANN
LMANN	Levenberg–Marquardt optimized ANN
GDANN	Gradient Descent optimized ANN
UCI	University of California, Irvine (Machine Learning Repository)
ML	Machine Learning
BP	Backpropagation
CNN	Convolutional Neural Network
GA	Genetic Algorithm
PSO	Particle Swarm Optimization
SCG	Scaled Conjugate Gradient
LM	Levenberg–Marquardt
GD	Gradient Descent
ACC	Metric of Accuracy
CE	Cross-Entropy
CK	Cohen’s Kappa
